# Individual differences in personality profiles among potential living kidney transplant donors

**Published:** 2013-12-31

**Authors:** Carlos J van-der Hofstadt, Jesús Rodríguez-Marín, Fermín Martínez-Zaragoza, Carlos de Santiago-Guervós

**Affiliations:** 1 Departamento de Psicología de la Salud. Universidad Miguel Hernándezcjvander@umh.es; 2 Unidad de Psicología Clínica de la Salud. Hospital General Universitario de Alicante, Spainrod.marin@umh.es; 3 Equipo de Coordinación de Trasplantes. Hospital General Universitario de Alicante, Spainsantiago_car@gva.es

**Keywords:** Living donors, kidney transplants, personality assessment

## Abstract

**Background:**

Although the psychological assessment of potential living kidney donors (PLKD) is part of the recommendations for action for any transplant coordination, there are not many studies that provide data about the importance of selecting donors for improving transplant outcomes. This work aims to raise awareness of potential kidney donors by designing methods for early detection of potential problems after the transplant, as well as by selecting the most suitable donors.

**Methods:**

This is a study of 25 PLKD drawn from the General University Hospital of Alicante. Participants completed the Millon Clinical Multiaxial Inventory (MCMI-III) for the study of personality characteristics.

**Results:**

Women scored higher than men in the compulsive personality scale, and individuals with a genetic link with the recipient scored higher on depressive and dependent scales than did those with other relationships (emotional or altruistic).

**Conclusions:**

Women showed a pattern of significantly more compulsive personality traits (cautious, controlled, perfectionist) within a non-pathological style. Among the PLKD, there were significantly more women, which is contrary to what typically happens with donations from cadavers. Genetically related subjects scored higher on depression than did those that were emotionally related. The personality assessment of candidates for PLKD can help with developing a post-transplant follow-up regimen for an improved quality of life.

## Abstract (es)

**Antecedentes:**

Aunque la evaluación psicológica de los donantes potenciales vivos de riñón (DPVR) forma parte de las recomendaciones de actuación de cualquier coordinación de trasplantes, sin embargo, no existen muchos trabajos que aporten datos sobre su importancia a la hora de seleccionar donantes o de mejorar los resultados del trasplante.

**Objetivos:**

Este trabajo tiene como objetivo el aumentar el conocimiento sobre los donantes potenciales de riñón a través del diseño de métodos para detectar precozmente posibles problemas después del trasplante, así como seleccionar a los donantes más idóneos.

**Métodos:**

Se presenta un estudio de 25 DPVR del Hospital General Universitario de Alicante. Los participantes completaron el Inventario Clínico Multiaxial de Millon (MCMI-III) para el estudio de sus características de personalidad.

**Resultados:**

las mujeres puntuaron más alto que los varones en la escala de personalidad compulsiva, y los individuos con un vínculo genético con el receptor puntuaron más alto en personalidad depresiva y dependiente que aquellos con otro tipo de relación (emocional o altruista).

**Conclusiones:**

Las mujeres presentaron un patrón de personalidad significativamente más compulsivo (prudente, controlado, perfeccionista), dentro de un estilo no patológico. Entre los DPVR, hay un número significativamente mayor de mujeres, contrariamente a lo que sucede en la donación procedente de cadáver. Los sujetos emparentados genéticamente puntúan más alto en depresión que los emparentados emocionalmente. La evaluación de la personalidad de los candidatos a DPVR, puede ayudar a programar un seguimiento postrasplante que mejoren su calidad de vida.

## Introduction

Kidney transplantation is currently the best treatment option for patients with chronic renal insufficiency that is found in the advanced stages of the disease. Also, when done with a living donor, the graft survival rate is higher due to the higher quality of the organ, and the reduced waiting time for the transplant^1^. Therefore the potential living donor can be considered as a fundamental part of this process.

Candidates for living donations have been classified into six types^2^: 1) genetically related to the recipient, 2) emotionally related to the recipient, 3) an altruistic direct relationship, 4) altruistic relationship but unrelated to the recipient 5) organ sellers, and 6) persons participating in ¨cross donation¨ programs when donation to the person with whom there is a relationship has not been possible to grant to the person, despite either a genetic or emotional relationship. In Spain, the law does not allow for the sale of organs, but the other options mentioned are possible.

To comply with regulations from the Transplant Coordination Center at the University General Hospital of Alicante, the living donor candidates were referred to the Clinical Psychology Health Unit to conduct a psychological evaluation of the subjects^3^.

 The transplant process that a potential living donor submits to, where surgery did not result in any physical benefit and, instead, later complications may have appeared, makes prior psychological evaluation of the candidate essential^4^.

Early studies came to regard even purely altruistic donators as being suspect of poor psychological health^5^; however, starting in the 60´s, most studies confirmed just the opposite, i.e. that most potential altruistic donors did not suffer from any mental illness^6^. The psychological evaluations carried out by the studies in this regard included a number of important areas, most notably mental health, in addition to understanding the risks and circumstances of the donation, an analysis of the potential donor-patient relationships, the motivation to donate, the decision to donate, and coping strategies before surgery and the possible subsequent complications, among others^7^ ^-^ ^11^. This assessment claimed to reveal if there were candidates who were contraindicated or who had limits to their donation, not only physical but psychological, as well. This evaluation has been widely reviewed in the literature which shows that among transplant programs there are a great variety of methods and criteria used for the psychological assessment - in some cases formal criteria were used, while in others, semi-structured clinical interviews were conducted^1^ ^,^ ^12^ ^-^ ^14^.

Studies that have addressed this issue focused on assessing the mental health of the individual by means of psychometric tests have used projective tests and alexithymia scales^15^, the Mini Mental Status Examination^16^ (MMSE), the Temperament and Character Inventory^16^ (TCI), the Minnesota Multiphasic Personality Inventory^5^ ^,^ ^17^ ^,^ ^18^ (MMPI) or the MMPI-2^16^. Also, the APA´s Diagnostic and Statistical Manual-IV (DSM-IV) has been used for such purposes^19^.

However, we found no data in the literature on the evaluation of PLKD by means of the MCMI-III. Previous studies have compared different versions of the MMPI and the MCMI as clinical assessment tools^20^ ^,^ ^21^ and indicated that the MCMI is also able to provide data on the patient's coping strategies in stressful situations. Moreover, it offers a perspective on the continuum between normality-abnormality in psychopathology which makes it particularly interesting for a collective assessment (of potential donors) that, in principle, does not assume some psychopathology.

## Objectives

This work has the objective of increasing awareness concerning potential kidney donors through the design of methods for early detection of potential problems following the transplant, as well as for selecting the most suitable donors in clinical terms.

## Materials and Methods

### Design

The work consisted of a correlation, cross-sectional, descriptive study. Differences were analyzed according to various personality variables, sex, marital status, educational level and the type of relationship between the donor and recipient. There were three categories considered for donor types that depended on the relationship with the recipient: genetically related, emotionally related to the recipient or indirectly altruistic. Also, scores were observed that ranged between 60 and 75 on the MCMI-III as clinical detection indicators of possible characteristic personality profiles.

### Procedures/ethical safety guards

Established protocols were followed for accessing data from the medical records of the Clinical Health Psychology Unit of the University General Hospital of Alicante for the publication of these research findings. From the Transplant Coordination Unit, candidates for living donorship signed an informed consent for conducting prior evaluation tests. Patients that were to be implanted also signed the informed consent for live donor nephrectomy for renal transplantation^22^. Next, the Transplant Coordination Unit sent potential donors to the Clinical Psychology Health Unit for evaluation. Evaluations were conducted by psychologists from the unit using established protocols and supportive testing. A first interview was conducted along with administration of the MCMI-III, and a second interview was performed for communicating the results. 

### Participants

The final sample consisted of 25 PLKD´s that were attended at the Transplant Unit of the University General Hospital of Alicante in the period between February 2009 and October 2011. The sample represents the total potential kidney donors in the province of Alicante during that period. The mean age was 43.80 years and it ranged from 30 to 55 years. 76% were women. Regarding marital status, it was found that 12% were single, 76% were married or living together and the remainder were either separated or divorced. 52% had a primary level education, 36% had a secondary education and the remainder reached the university level. Of the total participants, 13 had a genetic relationship with the potential recipient, 10 had an emotional relationship, and two were potential altruistic donors unrelated to the recipient. Among the initial sample of individuals, a woman was excluded as a candidate after presenting with an active psychopathology that could affect their ability to make the donation decision. Finally, among the individuals evaluated, 28% had the transplant performed, while 64% did not, 4% were taken from the cadaver and another 4% remained pending. In two cases involving males, the study was not completed at the request of the donor.

### Variables studied 

1) personality pattern, 2) pathological personality, 3) clinical syndromes of moderate severity, 4) severe clinical syndromes, 5) sex, marital status and educational level, 6) Relationship to the recipient: genetic, emotional and altruistic.

### Instruments

For variables 1-4, the Millon Clinical Multiaxial Inventory-III^23^ (MCMI-III) was used, which is currently a reference instrument for the clinical assessment of personality. This inventory consists of 175 items which are formatted as dichotomous, true-false responses, designed for use in clinical populations. It contains 11 clinical scales of personality patterns (schizoid, avoidant, depressive, dependent, histrionic, narcissistic, antisocial, aggressive, compulsive and negativistic), three pathological personality scales (schizotypal, borderline and paranoid), seven clinical syndromes of moderate severity (anxiety disorder, somatoform disorder, bipolar disorder, dysthymic disorder, alcohol dependence, substance dependence and PTSD), and three severe clinical syndromes (thought disorder, major depression and delusional disorder).

In all of the MCMI-III scales, scoring is set at 60 as the median obtained from all of the patients. For personality scales, scores between 75 and 84 suggest the presence of clinically significant personality features, while scoring at or above 85 is suggestive of a disorder. For clinical syndromes, scores between 75 and 84 indicate the presence of a syndrome, while scores of 85 or above denote the prominence of a particular syndrome. For variables 5 and 6, a socio-demographic data collection sheet was used.

## Results

Following the standard procedure for most psychometric tests, the validity data of the Millon test protocols were first checked and all were found to be valid.The data were analyzed using the SPSS program^24^. For statistical analysis of differences in terms of the relationship with the recipient, the Student t test for differences between means for independent samples was used, with a confidence interval of 95%. Comparisons were only made for the genetic and emotional relationship groups, since the altruistic relationship group was comprised of only 2 persons. Levene tests were made for checking the equality of inter-group variances as well as for the normal distribution of scores. Only the significant results will be reported. 

Persons genetically related to the potential recipient scored higher on depression (F Levene = 1.26, *p*= 0.273, t (21) = 2.06, *p*= 0.052) and dependency than did persons only related emotionally (F Levene= 0.321, *p*= 0.577, t(23)= 3.35, *p*= 0.082), marginally significant) Table 1.

**Table 1:**
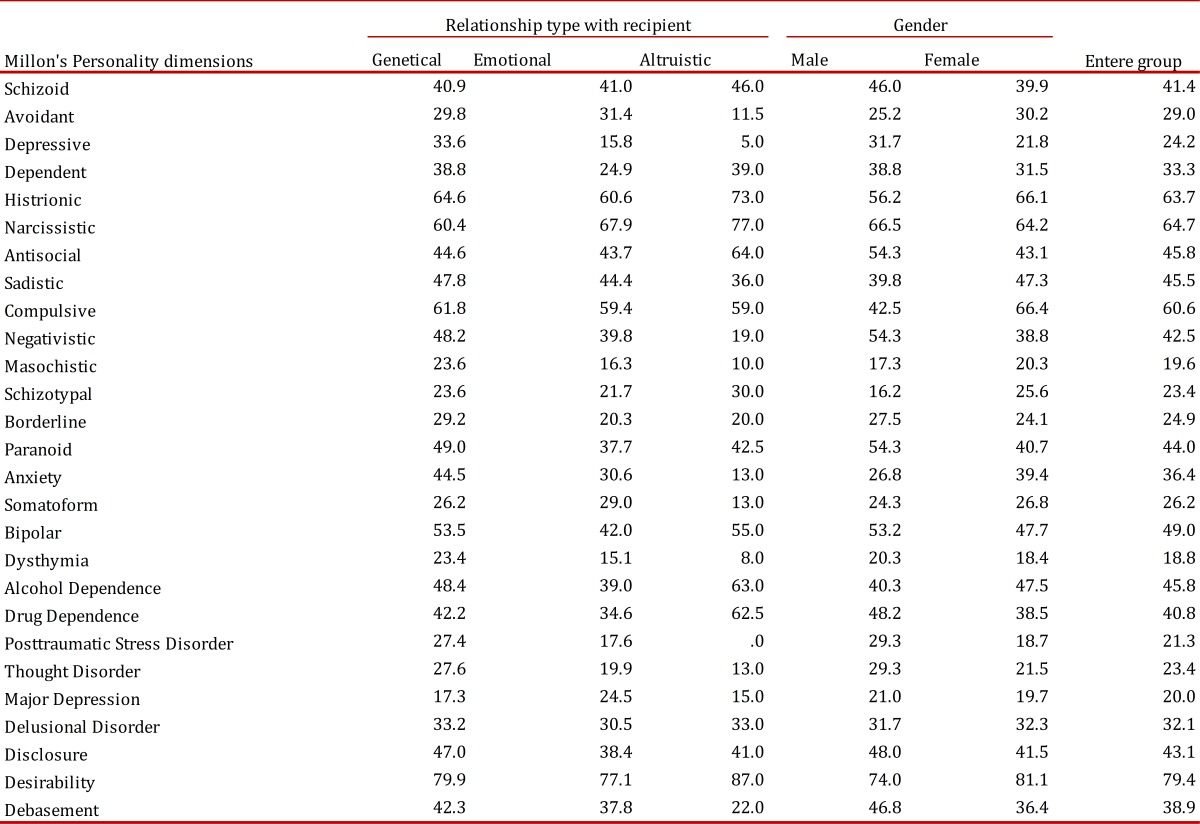
Personality differences according to the type of relationship with the recipient, sex and the entere group

With regard to the socio-demographic variables, significant differences were only found for the sex variable, after computing data for all of the participants. First, the normality of the distribution was checked resulting in a significant Kolmogorov-Smirnov test (*p*= 0.016; non-normal distribution). Thus, a nonparametric test was conducted (Mann-Whitney U). The results showed that women scored significantly higher on the compulsivity scale than did men (U= 12.50, *p*= 0.005) (Table 1).

The complete sample also scored an average of over 60 on histrionic, narcissistic and compulsive personalities, as well as on the scale of social desirability on the MCMI-III, in which the prevalence approaches 80. Only in some specific cases could one speak of personality disorders (Table 1).

## Discussion

The main objectives of this study were to examine whether psychological differences existed within the PLKD group and if these differences could lead to characteristic profiles. The results in this study would suggest that the candidates for PLKD showed different profiles according to sex and type of relationship with the recipient. As regards sex, women from the sample appeared to have a more compulsive personality pattern than did men, i.e., they could be described as cautious persons, controlled and perfectionistic, with a fear of social disapproval. We should note here that the relative frequency of the women´s group members when compared to the men´s group was greater, which is in contrast to data from the ONT^3^ - although the latter belongs to cadaveric transplants. Our sample of living donors seems to have a different demographic profile.

For its part, genetically related individuals show higher scores for depression when compared to emotionally related individuals, which is probably due to the different levels of emotional involvement. The average profile tends to score higher in the search for affection, approval and praise, and in overestimations of their own value.

There are no previous studies using the MCMI-III as a tool for the evaluation of potential donors; however, it has been done through other clinical instruments already mentioned.

Some authors^17^ administered the MMPI to a sample similar to that of PLKD and found that men had a more anxious profile (psychasthenia) and women had higher scores on social isolation. Our sample only indicates differences in compulsivity, an anxiety-related variable, but this was instead indicative of the women's group. On the other hand, the use of the MCMI-III presumes a change in conceptualization regarding personality-psychopathology relationships. However, the study by Rios-Martinez *et al*.^17^ did agree on the need for a social recognition of donors.

In the sample we have found a case in which the presence of psychopathology indicated a need for dismissal as a candidate for living donorship, which represents 4% of the total. This percentage is similar to that obtained in other studies^25^, related to subjects with an active pathology among candidates for PLKD. 

This result leads us to consider the usefulness of the assessment with regard to compliance with current regulations and the preservation of the health and rights of the donor.

The high scores on the social desirability scale are common to those found in the assessments made during selection processes and during evaluations of patients to be candidates for certain procedures and interventions (bariatric surgery, candidates for implant treatments in the Pain Unit, and assisted reproduction treatments), at least according to our clinical observation.

On the other hand, according to the test manual^23^, a frequent occurrence of prominent elevations in the histrionic, narcissistic and compulsive scales comes mainly in the absence of scores with significant elevations on scales of severe personality pathology and Axis I pathology (DSM-IV), which may reflect personality strengths as do moderate levels of self-esteem (narcissistic) or sociability (histrionic).

Should these results be taken into account when selecting a PLKD? Probably not, because there is no report on the necessary characteristics that allow for exclusion or inclusion itself for an individual, but we can talk about types of persons who present themselves as PLKD candidates, and this may help us to program follow-up with the PLKD that will improve psychosocial adjustment and quality of life after the transplant.

An important aspect of our study is that it uses a new measure of personality in which the person is not evaluated in terms of possible mental illnesses, but simply assumes that your personality is normal. This allows, when evaluated on a continuum, to make predictions of future problems in accordance with current ratings.

Further studies with a larger sample size should corroborate the results found in this study. It would also be similarly interesting to compare results with other well-regarded tests of personality assessment, such as the MMPI-2.
